# Impact of the somatosensory influence on annoyance and quality of life of individuals with tinnitus: A cross-sectional study

**DOI:** 10.1016/j.bjorl.2024.101542

**Published:** 2024-12-11

**Authors:** Wildna Sharon Martins da Costa, Lucas Barbosa de Araújo, Henrique de Paula Bedaque, Lidiane Maria de Brito Macedo Ferreira, Karyna Myrelly Oliveira Bezerra de Figueiredo Ribeiro

**Affiliations:** a Universidade Federal do Rio Grande do Norte, Programa de Pós-Graduação em Fisioterapia, Departamento de Fisioterapia, Natal, RN, Brazil; b Universidade Federal do Rio Grande do Norte, Departamento de Cirurgia, Natal, RN, Brazil

**Keywords:** Tinnitus, Somatosensory, Somatic, Quality of life, Self report

## Abstract

**Objective:**

To assess the impact of the somatosensory influence on annoyance and quality of life of individuals with tinnitus.

**Methods:**

This cross-sectional study included individuals with tinnitus of both sexes, aged ≥18 years, recruited from a specialized otolaryngology outpatient, and evaluated by a multidisciplinary team (otorhinolaryngologists, physical therapists, and audiologists). An evaluation adapted from the Diagnostic Criteria of Somatosensory Tinnitus (ST) was performed to verify the influence of the somatosensory system on the cause of tinnitus. The intensity of discomfort from tinnitus was assessed by the Numerical Rating Scale, the impact on quality of life by the Tinnitus Handicap Inventory (THI), and factors of exacerbation of tinnitus were self-reported. Individuals were divided into ST and non-ST groups. Unpaired *t*-test and Chi-Square were used for group comparisons; Cohen's *d* and Phi measured effect sizes.

**Results:**

A total of 100 individuals were evaluated: 46 from the ST group and 54 from the non-ST group. The ST group demonstrated greater discomfort from tinnitus (*p* <  0.01); stress was identified as a factor of exacerbation 82.6 (*p* <  0.01), and sleep disturbance as a consequence of tinnitus 63% (*p* =  0.04). Additionally, the ST group presented a greater impact on quality of life in the functional domain (*p* = 0.03) and total THI score (*p* =  0.05) than the non-ST group.

**Conclusion:**

Individuals with ST reported greater discomfort from tinnitus and a more significant impact on the quality of life.

## Highlights

• Individuals with somatosensory tinnitus experience higher annoyance.
• Stress and sleep disturbances are factors associated with the somatosensory tinnitus.
• The somatosensory influence causes a greater impact of tinnitus on quality of life.
• The somatosensory system must be considered when evaluating and managing tinnitus.

## Introduction

Tinnitus is the conscious perception of sound without an external auditory stimulus.^1^ Its prevalence in adults ranges from 5.1% to 42.7%^2^ and increases with age.^3^ In Brazil, the prevalence is 22.0%, affecting more women (26.0%) than men (17.0%).^4^ The variation in prevalence may be attributed to the multifactorial etiology of the symptom and the subjective nature of the evaluation.^3^ In addition, tinnitus can be subjective (perception of a sound without an external sound source) or objective (attributed to an internal sound source); acute (less than one month) or chronic (over three months); continuous or intermittent; and auditory (primary) or para-auditory (secondary).^5^

Somatosensory Tinnitus (ST) is caused by head, neck, jaw, or eye movements that change the auditory neural pathway via somatic action.^5^ The ST prevalence ranges between 16.0% and 83.0%;^6^, ^7^ 43.0% presents cervical origin.^7^ Also, individuals with tinnitus have a higher frequency of Temporomandibular Disorder (TMD),^8^ present in 64.0% of chronic tinnitus cases.^9^ Furthermore, the Temporomandibular Joint (TMJ) complaints increase from 19.0% in individuals with any type of tinnitus to 36.0% in individuals with severe tinnitus, suggesting a strong contribution of these TMJ complaints to the tinnitus severity.^10^

The tinnitus diagnosis considers the symptom complaint, a physical examination encompassing the diagnostic hypothesis, and audiological, laboratory, and imaging tests when indicated. The examination must also consider the presentation and characteristics of tinnitus, potential musculoskeletal influence, factors affecting the symptom, and the impact on quality of life.^11^ The perception of individuals on the impact caused by tinnitus varies according to their experience.^12^ Tinnitus impairs quality of life and may lead to cognitive impairments, memory lapses, concentration difficulties, and auditory alterations, which may interfere with leisure activities, rest, social and domestic environment, and emotional well-being (e.g., irritability, anxiety, depression, and sleep disturbance).^13^, ^14^, ^15^

Limbic structures (e.g., amygdala) are connected to the auditory pathway and are essential for emotional processing. They may also contribute to tinnitus.^16^ In the United States, about 10.0% to 20.0% of individuals with tinnitus report symptoms that reduce their quality of life,^17^, ^18^ and its occurrence is associated with suicidal ideation and attempts.^19^ A Hungarian study with 630 individuals with primary tinnitus revealed that 19.2% experienced a moderate and 14.2% a severe impact on quality of life.^20^ However, few studies reported the impact of the somatosensory influence on variables related to tinnitus. Thus, the present study aimed to assess the impact of the somatosensory influence on annoyance and quality of life of individuals with tinnitus.

## Methods

### Study design

This cross-sectional study was conducted by the Departments of Surgery and Physical Therapy of Federal University of Rio Grande do Norte from September 2021 to August 2023. The study followed the Strengthening the Reporting of Observational Studies in Epidemiology guidelines and was approved by the university research ethics committee under the number 4.471.809.

### Participants

Individuals were recruited from a specialized tinnitus outpatient in the otolaryngology department of the Onofre Lopes University Hospital. Eligibility criteria included individuals of both sexes aged ≥ 18 years reporting complaints of tinnitus. Those with cognitive deficits that could limit understanding and response to evaluative commands, who refused to participate, or who did not complete any evaluation were excluded. The Mini-Mental State Examination was used to assess cognitive aspects, and the cutoff points comprised educational level. To remain in the study, illiterate individuals were required to achieve a minimum score of 13 points; those with one to seven years of education, 18 points; and those with eight or more years of education, 26 points.^21^, ^22^ Scores below these thresholds indicated cognitive impairment and resulted in the exclusion of individuals from the study.

### Procedures

Individuals were assessed by a multidisciplinary team (otolaryngologist, physical therapist, and speech therapist) to screen the etiology of tinnitus. The otolaryngological evaluation included oroscopy, otoscopy, laryngoscopy, rhinoscopy, mastoid and temporal auscultation, and vestibular clinical examination. Complementary tests included biochemical evaluation, tonal and vocal audiometry, and tympanometry; the latter two were conducted by audiologists. The physiotherapeutic assessment, adapted from the diagnostic criteria for ST,^6^ detected the influence of the somatosensory system on the cause of tinnitus. The assessment was performed in a silent environment to avoid interference in tinnitus perception, following the same sequence for all individuals. The multidisciplinary team discussed the data to determine the cause of tinnitus. Influence on the somatosensory system and alterations in the auditory pathway were identified to divide all individuals into two groups for analysis: the ST group and the non-ST group.

### Assessment instruments

#### Diagnostic criteria for somatosensory tinnitus

The somatosensory influence on the origin of tinnitus was assessed using diagnostic criteria for ST,^6^ including tinnitus modulation, characteristics, and associated symptoms (Table 1). The modulation comprises changes (i.e., increases or decreases) in volume type of sound, location, or perception (constant or intermittent).

**Table 1:** Diagnostic criteria for somatosensory tinnitus.

**Criterion 1: Tinnitus modulation**
The individual is able to modulate tinnitus by voluntary movements of the head, neck, jaw, or eye (gaze-evoked)
The individual is able to modulate tinnitus by somatic maneuvers (isometric).
Tinnitus is modulated by pressure on myofascial trigger points.

**Criterion 2: Tinnitus characteristics**
Tinnitus and neck or jaw pain complaints appeared simultaneously.
Tinnitus and cervical or jaw pain aggravate simultaneously.
Tinnitus is preceded by a head or cervical trauma.
Tinnitus increases during "inappropriate postures".
Report of variation in pitch, loudness, and location of tinnitus.
In the case of unilateral tinnitus, the audiogram is normal.

**Criterion 3: Accompanying symptoms**
Tinnitus is accompanied by frequent pain in the cervical, head, or shoulder girdle.
Tinnitus is accompanied by the presence of myofascial trigger points.
Tinnitus is accompanied by increased muscle tension in the suboccipital muscles.
Tinnitus is accompanied by increased muscle tension in the extensor muscles of the cervical.
Tinnitus is accompanied by temporomandibular disorders.
Tinnitus is accompanied by teeth clenching or bruxism.
Tinnitus is accompanied by dental diseases.

#### Level of tinnitus annoyance

The level of tinnitus annoyance was measured by the Numerical Rating Scale, which varies from 0 to 10 and evaluates the perception of the individual on the symptom. Also, this scale is easy to understand and apply and has better psychometric properties than other scales.^23^, ^24^, ^25^ Individuals were asked: “On a scale from 0 to 10, where 0 corresponds to no discomfort from tinnitus, and 10 corresponds to the worst possible annoyance from tinnitus, what rating would you give today?”.

#### Impact of tinnitus on quality of life

The Brazilian version of the Tinnitus Handicap Inventory (THI) was used to assess the impact of tinnitus on quality of life.^26^ The questionnaire encompasses 25 questions divided into functional (11 questions), emotional (9 questions), and catastrophic (5 questions) aspects. Each question could be answered as no (0 points), sometimes (2 points), or yes (4 points). The total score is the sum of the points of each domain and ranges from 0 to 100 points. Higher scores indicate a greater impact of tinnitus on the quality of life.^26^, ^27^, ^28^ The impact can be classified as slight (0–16), mild (18–36), moderate (38–56), severe (58–76), and catastrophic (78–100).^29^

#### Self-reported factors of exacerbation of tinnitus and their influence on daily activities and sleep

The presence or absence of factors of exacerbation of tinnitus was verified using participant self-report by the following questions: “What factors worsen your tinnitus? What factors alleviate your tinnitus? Does your tinnitus impede the performance of daily activities? Does your tinnitus disrupt your sleep?”.

### Statistical analysis

Analyses were conducted using SPSS (version 23.0). The Kolmogorov-Smirnov test was used to assess data normality. A descriptive analysis was performed to characterize the sample, and results were presented using means and Standard Deviations (SD) or frequencies. The Chi-Square test, with effect size established by the *Phi* coefficient, was utilized to compare categorical variables between groups and self-reported factors of exacerbation of tinnitus and their influence on daily activities and sleep. *Phi* values classified the associations as very strong (>0.25); strong (>0.15); moderate (>0.10); weak (>0.05); or very weak (>0.00).^30^ The unpaired Student's *t*-test for independent samples was used to compare groups regarding THI scores, and Cohen's *d* was used to calculate the magnitude of differences between groups. Confidence Intervals of 95% were reported, and statistical significance was set at *p* < 0.05.

## Results

**Fig. 1:**
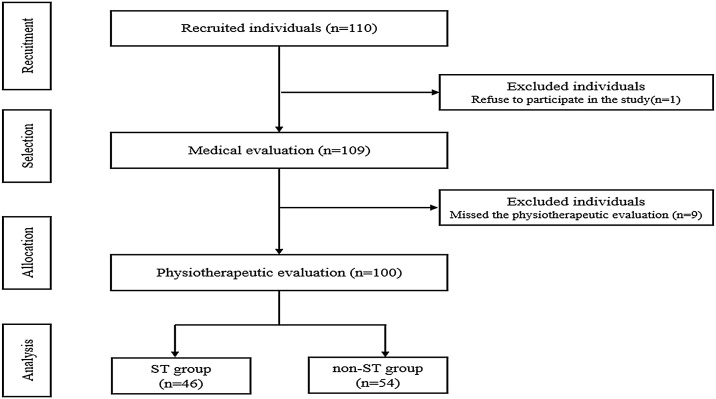
Study flowchart.

Of the 110 individuals with tinnitus screened for eligibility, 10 were excluded: one refused to participate and nine missed the physiotherapeutic evaluation. The study flowchart is illustrated in Fig. 1. The final sample comprised a total of 100 individuals; the ST group included 46 individuals, including exclusive ST diagnosis (*n* = 19) and mixed tinnitus with associated auditory alterations (*n* = 27), including hearing loss, presbycusis, tympanic membrane perforation, and eustachian tube dysfunction. The non-ST group included 54 individuals with tinnitus and without somatosensory influence, which 50 individuals presented various conditions, such as auditory causes, Meniere's disease, metabolic disorders, migraine, ototoxicity, concussion, otitis, neurological diseases, post-COVID, and anxiety, and four presented idiopathic causes.

Most were women (78.3% in the ST group and 63.0% in the non-ST group). Variables were not significantly different between groups (*p* >  0.05), except for age and otalgia. The mean age was higher in the non-ST group, reaching 58.02 (SD = 14.70). In addition, in 81.0% of the individuals who attended audiometry, hearing impairment was highly prevalent in both groups and was higher in the non-ST group (77.5%) than in the ST group 70.7% (*p* <  0.48). Regarding the perception of the side, 52.1% of the ST group and 63.0% of the non-ST group presented bilateral tinnitus. Sample characterization is presented in Table 2.

**Table 2:** Sample characterization (*n* = 100).

Variable	ST Group (*n* = 46)	non-ST Group (*n* = 54)	*p*
	Mean (DP) or F/RF (%)	Mean (DP) or F/RF (%)	
Age Mean (DP)	48.96 (17.9)	58.02 (14.7)	0.00^a^
Sex			0.09^b^
Women	36 (78.3)	34 (63.0)	
Men	10 (33.3)	20 (37.0)	
Time onset of tinnitus (months) Mean (DP)	51.5 (67.6)	74.2 (100.5)	0.19^a^
Onset of tinnitus			0.55^b^
Sudden	24 (52.2)	25 (46.3)	
Gradual	22 (47.8)	29 (53.7)	
Presentation of tinnitus			0.30^b^
Continuous	31 (67.4)	31 (57.4)	
Intermittent	15 (32.6)	23 (42.6)	
Pulsatile tinnitus			0.61^b^
Yes	6 (13.0)	9 (16.7)	
No	40 (87.0)	45 (83.3)	
Otological symptoms			
Hearing loss	25 (54.3)	35 (64.8)	0.28^b^
Otalgia	15 (32.6)	8 (14.8)	0.05^b^
Otorrhea	1 (2.2)	3 (5.6)	0.39^b^
Ear fullness	20 (43.5)	25 (46.3)	0.77^b^
Dizziness	29 (63.0)	34 (63.0)	0.99^b^
Comorbidities			
Yes	38 (82.6)	42 (77.8)	0.54^b^
No	8 (17.4)	12 (22.2)	
Use of ototoxic medications			
Yes	12 (26.1)	17 (31.5)	0.55^b^
No	34 (73.9)	37 (68.5)	
Side of tinnitus			
Right unilateral	12 (26.1)	10 (18.5)	0.71^b^
Left unilateral	10 (21.7)	10 (18.5)	
Bilateral	14 (52.1)	34 (63.0)	

F/RF (%), Absolute and Relative Frequencies; mean (SD), data presented as mean and Standard Deviation; ST, Somatosensory Tinnitus. Comorbidities: metabolic diseases, diabetes, hypertension, vestibular disorders, neurological diseases, accidents, traumas, and anxiety.

a p-Value of the Student's *t*-test.

b p-Value of the Chi-Square test.

The level of discomfort from tinnitus was significantly higher in the ST group (6.42 [SD = 2.40]) than in the non-ST group (4.94 [SD = 3.00]; *p* < 0.05). Moreover, individuals from the ST group reported stress 82.6% (*p* < 0.01) and sleep disturbance 63.0% (*p* =  0.04) as factors associated with tinnitus (cause and consequence, respectively). Phi values classification ranged from strong to very strong (0.20 to 0.28; Table 3).

**Table 3:** Self-reported factors of exacerbation of tinnitus and their influence on daily activities and sleep in ST and non-ST groups.

Variable	ST group (*n* = 46) F/RF (%)	non-ST group (*n* = 54) F/RF (%)	*p*	*Phi*
Stress			0.01^a^	0.28
Yes	38 (82.6)	30 (55.6)		
No	8 (17.4)	24 (44.4)		
Noise exposure			0.42	0.08
Yes	18 (39.1)	17 (31.5)		
No	28 (60.9)	37 (68.5)		
Consumption of stimulating foods			0.86	0.01
Yes	10 (21.7)	11 (20.4)		
No	36 (78.3)	43 (79.6)		
Impairment in daily activities due to tinnitus			0.08	0.17
Yes	24 (52.2)	19 (35.2)		
No	22 (47.8)	35 (64.8)		
Sleep disturbance due to tinnitus			0.04^a^	0.20
Yes	29 (63.0)	23 (42.6)		
No	17 (37.0)	31 (57.4)		

F/RF (%), Absolute and Relative Frequencies; p, p-value of the Chi-Square test.

a p-Value considered significant.

Tinnitus impacted more in the quality of life of the ST group, with significant differences observed in the functional aspect (*p* = 0.03) and total THI (*p* =  0.05); both variables presented medium effect sizes (0.43 and 0.51, respectively), as highlighted in Table 4.

**Table 4:** Impact of tinnitus on quality of life in emotional, catastrophic, and functional aspects of the Tinnitus Handicap Inventory between ST and non-ST groups.

THI aspects	ST group (*n* = 46)	non-ST group (*n* = 54)	*p*	Cohen's *d*
Emotional	17.95 (8.52)	15.37 (9.84)	0.16	0.28
Catastrophic	10.47 (4.73)	9.51 (5.24)	0.34	0.19
Functional	21.39 (10.74)	16.77 (10.55)	0.03^a^	0.43
Total score	49.82 (21.00)	41.29 (23.21)	0.05^a^	0.51

Data presented as mean and standard deviation. THI, Tinnitus Handicap Inventory; p, p-value of the Student's *t*-test.

a p-Value considered significant.

## Discussion

This study aimed to assess the impact of the somatosensory influence on annoyance and quality of life of individuals with tinnitus. Individuals from both groups were mostly women. Studies vary regarding the influence of sex on the prevalence of tinnitus; some indicate a higher prevalence in men,^31^, ^32^ women,^33^, ^34^ or propose no differences between sexes.^35^ The frequent use of healthcare services may justify the higher prevalence in women.^36^ Moreover, a study concluded that women rated higher levels of discomfort from tinnitus on the visual analog scale than men.^37^

In the present study, both groups presented a high percentage of individuals with auditory alterations and bilateral perception of tinnitus. These findings corroborate Biswas *et al*,^38^ who demonstrated that tinnitus is more prevalent in Eastern Europe, where 28.3% of people in Bulgaria experiencing tinnitus and 4.2% in Romania suffer from severe tinnitus, compared to Western Europe. This data may be related to a higher prevalence of hearing loss in these regions, as reported by the Global Burden of Disease study.^39^

Additionally, the non-ST group presented a higher mean age and prevalence of hearing loss. Hearing loss increases with age in individuals with tinnitus.^3^ A previous study indicated a frequency of 55.3% of hearing loss in individuals with tinnitus without somatosensory influence, 52.7% with somatosensory influence, and 45.0% with significant somatosensory influence,^40^ corroborating our results. Thus, hearing loss is associated with tinnitus and hinders communication, causing social isolation, increased prevalence of depression and anxiety, and cognitive decline.^41^

The ST group reported higher annoyance from tinnitus. A possible hypothesis is that ST includes anatomical and functional connections between the somatosensory and auditory systems,^42^ increasing the excitability of the trigeminal system.^43^ Moreover, individuals with chronic tinnitus and cervical pain present greater mechanical hyperalgesia in the trigeminal region and more symptoms indicative of central sensitization than those with only tinnitus or neck pain or healthy individuals.^44^

The ST group reported stress as a factor in the exacerbation of tinnitus and sleep disturbance as a consequence. Stress increases muscle contractions and is related to bruxism in this population.^45^, ^46^ Furthermore, individuals with tinnitus often suffer from sleep disturbances, even in milder cases.^47^ These conditions are a risk factor for developing depression and anxiety in this population; in addition, poorer sleep quality was correlated with higher scores on mental distress questionnaires.^48^ Moreover, insomnia and tinnitus share similar pathophysiological characteristics, such as activation of the limbic system and hyperactivation of the sympathetic autonomic nervous system,^48^ suggesting that managing sleep problems is important even in the early stages of tinnitus.^47^

The prevalence of psychiatric disorders (e.g., anxiety and depression) is high in individuals with tinnitus and is associated with increased levels of annoyance or severity (or both).^10^, ^18^, ^49^ Previous studies did not report the high prevalence of anxiety and stress in individuals with and without ST;^50^ however, both conditions may negatively impact the severity of tinnitus in individuals with ST.^46^ Furthermore, severe or clinically significant tinnitus has been strongly associated with anxiety and depression.^10^, ^49^

The ST group presented a higher impact on quality of life, with higher scores in the functional aspect and THI total score. The use of THI during the diagnostic and therapeutic process helps the self-perception of individuals by quantifying the emotional, functional, and catastrophic impact of tinnitus on quality of life.^51^ Therefore, the THI increases the reliability of results.^51^, ^52^ According to this scale, individuals with mild tinnitus often present sleep disturbances and hearing difficulty. Additionally, individuals with moderate THI scores report increased discomfort perception, expressed by fatigue, depression, anxiety, and difficulty in enjoying life. Thus, more severe tinnitus corresponds to a higher probability of comorbidities associated with the symptom.^47^

As a limitation, the study did not include a control group of healthy individuals, which may lead to potential biases, and subgroup analysis based on different regions of somatosensory influence, such as the cervical, temporomandibular region, or both. Nevertheless, previous studies did not evaluate the tinnitus annoyance and quality of life associated with the presence (or not) of the somatosensory influence. Also, the evaluation conducted by a multidisciplinary team allowed an accurate diagnosis of the etiology of tinnitus and proper referrals.

## Conclusion

Individuals with ST reported greater annoyance, stress as a factor of exacerbation, and sleep disturbance as a consequence of tinnitus. Additionally, individuals with ST presented a greater impact of this symptom on quality of life than non-ST. The influence of the somatosensory system on the onset or exacerbation of tinnitus is important for proper referrals.

## Funding

This study was financed in part by the *Coordenação de Aperfeiçoamento de Pessoal de Nível Superior* – Brasil (10.13039/501100002322CAPES) – Finance Code 001.

## Conflicts of interest

The authors declare no conflicts of interest.
